# Unmasking the ties of snake bite poisoning and COVID-19

**DOI:** 10.1186/s42077-022-00256-9

**Published:** 2022-07-28

**Authors:** Anjuman Chander, Tanvir Samra, Sekar Loganathan, Varun Mahajan

**Affiliations:** Department of Anaesthesia, Post Graduate Institute of Medical Education and Research, Sector 12, Chandigarh, 160012 India

**Keywords:** Snake bite, COVID-19, ASV, Coagulopathy

## Abstract

**Background:**

Snake bite envenoming is a neglected tropical disease with variable clinical presentation, neurotoxic manifestations (respiratory paralysis), rhabdomyolysis, cardiotoxicity, autonomic hyperactivity, and/or coagulation abnormalities. There is limited data on the clinical course of the envenomation in an incidentally diagnosed COVID-19 patient.

**Case presentation:**

A 17-year-old male with history of snake bite and neuroparalysis developed shortness of breath. He was treated with lyophilized polyvalent anti-snake venom (ASV) on admission in the emergency department and mechanical ventilation. Subsequently, he tested positive for COVID-19 infection. No immunomodulatory therapy was administered, and patient was extubated on the 5th day of ICU admission without any neurological deficit.

**Conclusions:**

Coinfections of the severe acute respiratory syndrome coronavirus-2 (SARS-CoV-2) virus with snake bite poisoning lead to diagnostic dilemmas and controversies in management practices. Abnormalities of coagulation need to be cautiously addressed, and cause of development of pneumonia needs to be identified. The rapid recovery of the patient in our case theoretically can be explained on the organ-protective potential of snake-derived peptides; a large case series is however needed to prove the same.

**Supplementary Information:**

The online version contains supplementary material available at 10.1186/s42077-022-00256-9.

## Background

Coinfections of the severe acute respiratory syndrome coronavirus-2 (SARS-CoV-2) virus with other respiratory viruses and endemic diseases lead to diagnostic dilemmas and controversies in management practices (Burrel et al. 2021; Contou et al. 2020; Radisic et al. 2020). India has approximately 216 snake species, and patients with snakebite poisoning tested positive for SARS-CoV-2 virus are not only an additional healthcare burden during COVID-19 pandemic but a treatment challenge as well (Bawaskar et al. 2017; Moos 2020). The propensity of both snake envenomation and COVID-19 to cause an immune dysregulation labeled as “cytokine storm” characterized by constitutional symptoms, systemic inflammation, and multiorgan dysfunction is the most feared complication and an indicator of poor prognosis (Fajgenbaum and June 2020). Exploitation of the organ-protective potential of snake-derived bradykinin-potentiating peptide (BPP-10c), which has the potential to decrease angiotensin II levels, increases bradykinin-related effects on the bradykinin-2 receptor, and nitric oxide-mediated effects for developing an anti-SARS-COV-2 drug are a theoretical hypothesis which will also be discussed in this case report (Gouda and Mégarbane 2020a).

## Case presentation

A 17-year-old male with history of snake bite on upper back while sleeping on the floor and who developed bilateral ptosis and shortness of breath (respiratory rate 24/min, SPO2 87% on room air) was admitted in the emergency department during COVID-19 pandemic. On examination, there were double puncture marks approximately 15 cm below nape of the neck. The patient was hemodynamically stable (*HR*, 90/min; *BP* = 120/90 mm Hg). Lyophilized polyvalent anti-snake venom (ASV) was administered in an initial dose of 10 vials administered via intravenous route over a period of 1 h followed by a repeat dose of 10 vials after an hour. Oxygenation was provided with a Ventimask (*FiO2* = 0.5), but the patient developed acute type 2 respiratory failure after 2 h and was subsequently intubated after preoxygenation and use of sedative drugs like propofol (Saini et al. 2014).

In line with our institutional protocol, the patient was tested for SARS-CoV-2 virus using real-time reverse transcription-polymerase chain reaction (RT-PCR) assay after admission in the medical emergency department. He was shifted to the COVID ICU for mechanical ventilation and supportive care when reported as positive for COVID-19 infection.

To monitor progression of both pathologies, various biochemical investigations were performed on daily basis (Table 1). Hemogram, electrolytes (sodium, potassium, chloride, calcium, magnesium), renal function test, total proteins, albumin, liver function test, procalcitonin, lactate dehydrogenase (LDH), lactate, pro-BNP, CKMB, and trop T were normal. Blood, urine, and tracheal cultures were sterile, but piperacillin and tazobactam (4.5 G QID) were started empirically after admission. A chest CT examination was deferred, and CXR was used for monitoring as the patient did not develop acute respiratory distress syndrome (ARDS).

**Table 1:** Result of biochemical investigations

Days in ICU	1st	2nd	3rd	4th	5th	6th	7th
**TLC (cells/mm**^**3**^**)**	13,200	12,900	13,600	10,000	10,300	13,500	16,000
**Neutrophils%**	88.2	87.6	84	87	79	76	79
**Lymphocyte%**	5.4	5.6	5.6	2.7	6	4.8	5.7
**NL ratio**	16.33	15.64	15	32.2	13.1	15.8	13.8
**D-dimer (0–240 ng/mL)**	284	173	565	582	1158	1851	2142
**Fibrinogen (2–4 g/L)**	4.04	3.4	6.3	9.68	7.23	8.8	11
**Platelet (× 10**^**9**^**/L)**	310	306	218	267	288	235	433
**PT (10.4–14.8 s)**	15.8	15	18	16.4	16	16.5	18
**PTI (80–100%)**	88	88	76	85	85	84	75
**INR**	1.13	1.1	1.3	1.1	1.1	1.1	1.3
**APTT (26–35 s)**	27.2	56	51	30	32	32	30
**Ferritin (30–400 ng/mL)**	61	176	260	302	-	314	
**CRP (0–5 mg/L)**	61.27	180	353	333	230	191	205
**Mechanical ventilation**	SIMV 5/12	SIMV 5/12	CPAP 5/8	SIMV 5/10	SIMV 5/8	NIV	NIV
**FiO2**	0.4	0.4	0.4	0.4	0.4	0.7	0.5
**PaO2/FiO2 ratio**	172	166	160	180	190	234	250

*TLC* total leucocyte count, *NL ratio* neutrophil lymphocyte ratio, *PT* prothrombin time, *PTI* prothrombin time index, *INR* international normalized ratio, *APTT* activated partial thromboplastin time, *CRP* C-reactive protein, *FiO2* fraction of inspired oxygen, *Pao2* partial pressure of oxygen, *SIMV* synchronized intermittent mandatory ventilation, *CPAP* continuous positive airway pressure, *NIV* noninvasive ventilation

Enoxaparin (0.4 ml SC BD) and dexamethasone (6 mg BD) were administered. Over the course of next 3 days, patients GCS improved to 12/15 with (eyes 4, voice 2, motor 6), and the patients ventilatory support was shifted from synchronized intermittent mandatory ventilation (SIMV) to pressure support. After assessing clinical and arterial blood gas parameters, he was subsequently extubated on the 5th day after testing for bulbar reflexes and given intermittent noninvasive ventilation for 2 additional days. He was tested negative for SARS-CoV-2 virus after a week, and the patient was discharged home after 13 days of hospitalization with no neurological deficit. The patient was reviewed in outpatient clinic 4 and 12 weeks later. He had returned to work after 4 weeks of discharge. There was no residual pain or weakness.

## Discussion

Snakebites can cause a broad spectrum of symptoms with variable severity depending on the species of the snake, dose of venom injected, anatomic location of bite, age, health, size, and immune status of the victim (Warrell 2010). Coinfection with SARS-CoV-2 and its symptomatology adds to the heterogeneity in the clinical presentation of snake bite patients. Four main points of consideration in this subset of patients are as follows:

- *Firstly*, differentiation of venom-induced consumption coagulopathy (VICC) occurring in envenomization by viperid snakes, certain elapids, and a few colubrid (rear fang) snakes from COVID-19-associated coagulopathy/DIC (Jeon et al. 2019) is a challenge. VICC is characterized by prolonged INR, hypofibrinogenemia, thrombocytopenia, and increased fibrin degradation products which autocorrects with the administration of ASV (Stone et al. 2013). COVID-19 is characterized by elevations in fibrinogen and D-dimer levels, mild prolongation of PT/aPTT, and mild thrombocytopenia (platelet count ~100 × 10^9^/L) (Bikdeli et al. 2020). Thromboprophylaxis with LMWH is recommended for all hospitalized COVID-19 patients unless there is active bleeding, platelet count less than 20–30 × 10^9^/L, or fibrinogen less than 0.5 g/L (Barnes et al. 2020). But guidelines for anticoagulation in COVID-positive patients with snake bite have not been developed.
- *Secondly*, etiology for the development of pneumonia in intubated COVID-positive snake bite patients could be due to aspiration from bulbar paralysis, development of ventilator-associated pneumonia, or COVID-19 pneumonia.
- *Thirdly*, ambiguity persists in the use of immunomodulatory therapy (tocilizumab) in snake bite patients with local inflammation, secondary bacterial infection, or ventilator-associated pneumonia as its use has the potential to aggravate inflammation and lead to cellulitis or widespread sepsis.
- *Fourthly*, the cellular immune responses triggered by COVID-19 develop through the overexpression of CD8 and hyperactivation of cytotoxic T lymphocytes. Naja naja atra venom (NNAV) and cobrotoxin restore the CD4/CD8 ratio and have lung-protective properties by inhibition of lung inflammation, thrombosis in both arteries and veins, and attenuation of the development of fibrotic lesions in the lung (Gouda and Mégarbane 2020b). Naja naja atra venom (NNAV) and α-neurotoxins have broad-spectrum antiviral activities and thus a potential to be developed as an alternative therapy for COVID-19 (Lin et al. 2020).

## Conclusions

Abnormalities of coagulation need to be cautiously addressed, and cause of development of pneumonia needs to be identified. Administration of immunomodulatory therapy (Tocilizumab) for COVID 19 infection in a snake bite victim is a relative contra-indication. The therapeutic potential of venom in this subset of patients needs to be explored.

## Supplementary information

**Additional file 1.**

## Data Availability

Data is available from the corresponding author on reasonable request.

## Abbreviations

ICU: Intensive care unit
NNAV: Naja naja atra venom
SIMV: Synchronized intermittent mandatory ventilation
VICC: Venom-induced consumption coagulopathy
ASV: Anti-snake venom
RT-PCR: Real-time reverse transcription-polymerase chain reaction
LDH: Lactate dehydrogenase
ProBNP: Pro b-type natriuretic peptide
CKMB: Creatine kinase-MB
Trop T: Troponin T
DIC: Disseminated intravascular coagulation
