# Supplementary material for: Unmasking the ties of snake bite poisoning and COVID-19
Source: Ain-Shams J Anesthesiol. 2022 Jul 28;14(1):58. doi: 10.1186/s42077-022-00256-9 (PMC9330936; doi:10.1186/s42077-022-00256-9)
Supplement: Supplementary file 1 — Additional file 1. [file 42077_2022_256_MOESM1_ESM.docx]

| Sl. no | **Editor/reviewer Comments** | **Clarifications** |
| --- | --- | --- |
| 1.  2  3  4  5  6  7  8  9 | Please combined Ethics approval statement and consent to participate statement.  Please rename Ethics approval to Ethics approval and consent to participate  Please confirm whether the informed consent obtained from study participants was written or verbal, and clearly state this in your manuscript in the 'Ethics approval and consent to participate' subsection of the 'Declarations'. If verbal, please state the reason and whether the ethics committee approved this procedure. If the need for consent was waived by an IRB or is deemed unnecessary according to national regulations, please clearly state this, including the name of the IRB or a reference to the relevant legislation.  Please confirm in the ‘Consent for publication’ statement whether written informed consent to publish this information was obtained from study participants. Please be advised that proof of consent to publish from study participants can be requested at any time.  Please update your Competing interests  Please update your Availability of data and materials  Please include a statement in the Authors' contributions section to the effect that “all authors have read and approved the manuscript”, and ensure that this is the case.  Kindly move Cover letter after Declaration section in Title page.  Please remove Editor/reviewer Comments in a separate file, Please upload as a “Supplementary Material” file. | All corrections have been made on the last page of the manuscript |
|  |  | It has been moved  Uploaded separately |
